# What is drier? Understanding humidity in green-certified dwellings: a winter case study from Auckland, New Zealand

**DOI:** 10.1080/03036758.2025.2463450

**Published:** 2025-02-19

**Authors:** Rochelle Ade, Michael Rehm, Vishnupriya Vishnupriya

**Affiliations:** a Department of Property, University of Auckland Business School, Auckland, New Zealand; b School of Built Environment, Massey University, Auckland, New Zealand

**Keywords:** Green building rating tools, green building, absolute humidity, indoor environment quality, relative humidity

## Abstract

Despite the growing emphasis on green-certified buildings, there remains a notable gap in understanding their humidity performance, particularly in residential settings. This study addresses this gap by evaluating the wintertime humidity performance of 40 subsidised, 7-Homestar certified apartments for older residents in Auckland, New Zealand. While current building science guidelines recommend an optimal relative humidity (RH) range of 40% to 60%, our results show frequent exceedances of this range. However, when assessed using absolute humidity (AH), the apartments consistently fell within the epidemiologically acceptable range of 8–14 g/kg. This discrepancy highlights the need to reconsider which humidity metric—RH or AH—better reflects acceptable indoor environmental quality for health and comfort. Resident feedback indicated satisfaction with indoor humidity levels, despite elevated RH. This study calls for a reassessment of humidity guidelines in green-certified buildings to better balance occupant health, comfort, and energy efficiency.

## Introduction

Certified green buildings are frequently promoted as warmer, drier and healthy than non-certified buildings. For example, the New Zealand Green Building Council (NZGBC) states ‘Building a Homestar rated home helps to ensure a healthier, warmer, drier, and more comfortable home that uses less power and water than a similar home built to the minimum standard of the New Zealand Building Code’ (NZGBC 2024).

While ‘warmer’ is straightforward to define as higher temperature, the term ‘drier’ is more complex, as its meaning depends on the context. In building performance science, ‘drier’ typically refers to reduced moisture, which encompasses a decrease in water vapour in the air (measured through absolute humidity) or moisture content in materials. In contrast, ‘dampness’ specifically refers to unwanted moisture accumulation, often in building materials, indicating a persistent moisture issue.

Although related, dampness and humidity are distinct concepts and should not be used interchangeably. The Oxford Dictionary defines dampness as ‘the state or condition of being slightly wet’, whereas humidity refers to ‘the quantity of water vapor in the atmosphere’. Humidity describes an atmospheric condition, while dampness is a physical condition within a building that can result in problems such as mould growth and material degradation.

High humidity can contribute to dampness when water vapour condenses on cooler surfaces (e.g. windows or walls), leading to water accumulation. However, dampness can also occur independently of high humidity due to water infiltration, such as leaks or rising groundwater. So, how do you define ‘drier’ in the context of a ‘*healthier, warmer, drier, and more comfortable home’* (NZGBC 2024)?

### What is ‘drier’?

The concept of ‘drier’ in the context of building performance can refer to multiple factors, including:

- Reduced relative humidity (RH): controlling ambient RH to improve comfort and reduce the risk of condensation and biological growth (Sterling et al. 1985).
- Absolute humidity (AH) control: reduced viral transmission (Qi et al. 2022), condensation risk and material degradation (Overton 2019).
- Condensation control: preventing moisture buildup on surfaces, particularly windows and walls, to avoid damage and discomfort Overton (2019).
- Leak and infiltration control: fewer sources of water intrusion into a building, such as through leaks or unsealed gaps (Rehm 2009).
- Reduced interstitial moisture: reducing the amount of water trapped in building components, preventing issues like interstitial moisture (moisture accumulating between layers of a wall or roof assembly) (Künzel 2015).
- Minimisation of growth of biotic agents: limiting mould, mildew, and dust mites, which thrive in damp environments and can harm indoor air quality (Sterling et al. 1985).

RH is often used as a proxy for ‘drier’ environments in building science because it directly influences occupant comfort, condensation, and biological growth risks. However, AH complements RH by directly impacting viral transmission, material behaviour, and energy performance.

In summary, humidity (RH and AH) measures the moisture content in the air, whereas dampness (e.g. condensation, leaks, interstitial moisture) refers to moisture accumulation on or within building materials. Therefore, ‘drier’ can be understood as a holistic concept encompassing both humidity control and dampness mitigation strategies within the building envelope.

## Literature Review

### Green building rating tools

Most green building rating tools do not explicitly define dryness or dampness, nor do they establish specific humidity targets. Instead, these aspects are typically addressed indirectly through standards such as American Society of Heating, Refrigerating, and Air-Conditioning Engineers (ASHRAE) Standard 55 and Chartered Institution of Building Services Engineers (CIBSE) Guide A (thermal comfort) and ASHRAE 62.1 (ventilation). There are exceptions, as summarised in Table 1:

- LEED (Leadership in Energy and Environmental Design) ^1^ includes a moisture load control credit, which requires dehumidification equipment with sufficient latent capacity to maintain relative humidity (RH) at or below 60%.
- WELL Building Standard (WELL) offers two options:
  1. Mechanical humidity control systems capable of always maintaining RH between 30% and 60% by adding or removing moisture.
  2. Long-term humidity data demonstrating that RH levels in regularly occupied areas (excluding high-humidity spaces) remain between 30% and 60% during occupied hours.^2^

**Table 1.** Performance requirements of green building rating tools.

LEED	WELL	Homestar
*BD + C:Homes v3(2008)*	*v2*	
RH <60%	>30% RH <60%	–

These requirements reflect the importance of controlling humidity to ensure occupant comfort, mitigate condensation, and manage biological growth risks.

Unlike LEED and WELL, Homestar does not include specific humidity performance thresholds or ranges, possibly reflecting the more naturally ventilated nature of New Zealand housing. Instead, Homestar has historically adopted a more prescriptive control-based approach, which has evolved across its versions:

- **Version 3**:

The focus was on moisture source control, condensation minimisation, and ventilation. Requirements included:

1. Minimising moisture sources (e.g. overflows, ground covers under suspended timber floors, flues for gas heaters, and fully enclosed showers).
2. Reducing condensation through specified R-values for building elements.
3. Providing localised ventilation at moisture sources, such as kitchen rangehoods and bathroom/laundry extraction.
4. Ensuring moisture removal on a whole-house scale, either through an openable window area (WWR) of 5% of total floor area or mechanical supply ventilation.

- **Version 4**:

The focus shifted toward improved thermal performance and air tightness (whilst still maintaining the version 3 ventilation requirements). Additional requirements included:

1. Minimising thermal bridges using specified R-values for building elements and/or thermally broken window frames.
2. Reducing condensation through ground covers, air and vapour control layers, and air leakage testing.

- **Version 5 (current)**:

For the first time, Homestar mandates the use of whole-house, continuous mechanical ventilation for moisture control. Key requirements include:

1. Ventilation strategies, such as continuous extract, balanced, or balanced systems with heat recovery.
2. Minimising condensation on surfaces through thermal bridge fRsi temperature factors.
3. Minimising moisture in the building envelope using ground covers, air and vapour control layers, and air leakage testing.

This progression highlights Homestar's increasing emphasis on ventilation, thermal performance, and moisture control to improve indoor environmental quality and building durability. Tables 1 and 2 highlight a notable distinction between rating tools, with LEED and WELL relying on performance-based, RH (i.e. building science) measures with no AH (i.e. epidemiological) metrics or controls, while Homestar adopts prescriptive methods.

**Table 2.** Prescriptive (moisture control) requirements of Homestar.

		Homestar		
		*v3*	*v4*	*v5*
Prescriptive measure	Overflow	✓		
	Ground cover	✓	✓	✓
	WWR >5%	✓	✓	
	rangehood & bathroom/laundry (intermittent extract)	✓	✓	
	rangehood & bathroom/laundry (continuous extract)			✓
	Minimum R-Values		✓	✓
	fRSI factors			✓

The RH 60% threshold used by LEED and WELL has an interesting historical basis. Up until 1981 ASHRAE prescribed an acceptable RH range of 20% to 60% (ASHRAE 1966; Psomas et al. 2021). However, following the 1970s oil crises, ASHRAE raised the upper limit to 90% RH in 1981 to allow for greater energy conservation (Sterling et al. 1985). By 1985, Sterling et al. advocated for narrowing the acceptable range back to 40% to 60% RH, citing concerns over indoor air quality and occupant health. The primary aim was to mitigate the growth of biological pollutants, such as bacteria, viruses, and mould, while maintaining occupant comfort—despite the resulting increase in energy use.

Subsequent research, including work by Künzel (2015), further supported this range, concluding that maintaining indoor RH between 40% in winter and 60% in summer is generally safe for living spaces, including moisture-prone areas like kitchens and bathrooms.

This historical evolution underscores the balance between energy efficiency, indoor air quality, and occupant health in humidity management. However, Künzel’s research focused primarily on hygrothermal performance, examining how interior RH affects the moisture behaviour of building envelopes. It highlighted the importance of adequate ventilation to control indoor humidity and maintain moisture balance, emphasising that excessive indoor humidity—particularly in well-insulated buildings—can lead to condensation within walls and long-term structural damage.

Unlike RH, the literature lacks consensus on an acceptable AH range for indoor environments, with limited research available in residential contexts. Recent studies have explored AH’s role in viral transmission, particularly for SARS-CoV-2. For example:

- Bukhari et al. (2020) found that 85% of COVID-19 cases occurred at outdoor AH levels between 1 and 9 g/m³.
- Sajadi et al. (2020) reported significant transmission occurring within the 4–7 g/m³ range.
- Jansson and Yamamoto (2022) associated elevated transmission risk with higher AH levels exceeding 15 g/kg.
- Qi et al. (2022) observed a U-shaped relationship between weekly average AH and influenza activity, identifying minimised transmission risk around 12 g/m³ in Shanghai, a climate comparable to Auckland’s.

Synthesising these findings suggests that an AH range of 8–14 g/m³ may capture both the lower and higher humidity levels associated with increased infection risk. However, there is currently no established consensus on this range.

Furthermore, older individuals are more susceptible to illnesses due to weakened immune systems and reduced vaccine effectiveness caused by immunosenescence (Tanner et al. 2021). Research also suggests that older occupants may require higher humidity levels for thermal comfort compared to younger individuals. For instance, while ASHRAE recommends a minimum winter RH of 30%, this may not be sufficient for older people and could contribute to issues such as dry skin (White-Chu and Reddy 2011). Jin et al. (2020) further emphasised the importance of considering age-specific humidity needs for thermal comfort in indoor environments.

This evidence highlights the need for more nuanced humidity guidelines that account for both occupant demographics and the potential health impacts of RH and AH in residential settings.

### Humidity performance of certified residential green buildings

Despite the limited number of green building rating tools that include humidity ranges or thresholds, research on humidity performance in residential, green-certified buildings remains scarce, with few studies specifically addressing this metric (see Table 3).

**Table 3.** Humidity performance of certified green residential buildings.

Study	# of buildings	Country	Humidity findings	Green Rating
Akom et al. 2017	4	Canada	RH 33% to 43%	LEED
Alborz and Berardi 2015	1	USA	Lowest RH of 15.5%	LEED
Gupta et al. 2019	undisclosed	India	undisclosed	undisclosed
Su et al. 2021	16	China	RH 75% to 83% (summer)	undisclosed
Ade and Rehm 2022	30	New Zealand	RH 25% to 85% (summer)	Homestar
Ade and Rehm 2021b	30	New Zealand	RH 57% to 70% AH 9.5–11.2 g/kg (winter)	Homestar

Two studies in North America have reviewed the humidity performance of LEED-certified dwellings. In Canada, four LEED-certified houses recorded humidity levels between RH 33% and RH 43% (Akom et al. 2017). In the United States, a LEED-certified university hall of residence reported humidity as low as RH 15.5%, falling below ASHRAE Standard 55 and potentially posing health risks (Alborz and Berardi 2015). Notably, LEED does not include specific mandatory humidity benchmarks.

In India, an assessment of green-rated housing found similar RH levels in flats with and without air conditioning, though exact results were not provided (Gupta et al. 2019). Meanwhile, a study in Dalian, China, revealed high indoor RH levels (75–83%) in a certified public building. Interestingly, only 1.9% of occupants perceived the environment as humid, suggesting a potential tolerance for high humidity in coastal areas (Su et al. 2021). Across these studies, humidity performance has been a secondary focus, typically treated as part of broader thermal comfort assessments, and none of the studies reported absolute AH results.

In New Zealand, recent studies by Ade and Rehm (2020, 2021a, 2021b) have investigated indoor environmental quality performance in green-certified residential dwellings in Auckland. By comparing three housing types—older homes, newly constructed homes, and newly constructed, green-certified homes—they examined moisture performance using RH, AH, dew point depression, and predicted dust mite growth potential.

The literature reveals a fundamental lack of understanding among both academics and industry practitioners regarding the definition of ‘drier’ within the context of green building rating tools. This ambiguity may explain the limited research in this area, particularly with respect to ambient humidity, as few appear to fully grasp what is being measured—whether RH, AH, or both.

When considering prescriptive moisture control measures, such as the 5% operable window area requirement in Homestar v4, it is essential to account for correlations between interior and exterior conditions. This relationship is particularly significant in naturally ventilated buildings, which rely on outdoor air to maintain indoor air quality and thermal comfort (Mushore et al. 2017). However, in climates like Auckland, New Zealand, where outdoor air is often saturated with moisture, natural ventilation may provide minimal benefit in reducing indoor humidity levels.

### Correlation between indoor and outdoor environments

Without mechanical dehumidification, indoor humidity levels are expected to largely mirror outdoor conditions, fluctuating in response to external changes. While temperature typically demonstrates a linear correlation with outdoor conditions, particularly at higher temperatures (Nguyen et al. 2014; Pan et al. 2021; Yang and Lei 2022), relative humidity (RH) exhibits a weaker correlation (Lee and Lee 2015; Nguyen and Dockery 2016; Pan et al. 2021). In contrast, absolute humidity (AH) often shows a stronger positive linear correlation across all temperatures and seasons (Nguyen et al. 2014; Nguyen and Dockery 2016; Quinn and Shaman 2017; Wolkoff 2018).

This trend may arise because indoor RH tends to follow a more seasonal pattern compared to outdoor RH, with higher values typically observed in summer and lower values in winter, especially in subtropical regions. Conversely, outdoor AH exhibits less pronounced seasonal variation (Yang and Lei 2022).

### Summary

Although green building rating tools such as Homestar claim to deliver ‘drier’ performance outcomes, they often lack a clear definition of the term and fail to establish specific performance standards or measurable metrics to evaluate these claims. This study addresses this gap by contributing to the limited understanding of post-occupancy humidity levels in certified green residential buildings. It examines a 7-Homestar (v4) Built certified apartment building providing subsidised rental housing for residents over 65 in Auckland, New Zealand.

## Data and methods

The study focuses on a 40-unit apartment building in Auckland, New Zealand. The building accommodates residents aged 65 and older, including both single occupants and couples. Self-reported occupancy patterns range from full-time presence to part-time occupancy (∼50%).

**Figure 1.**
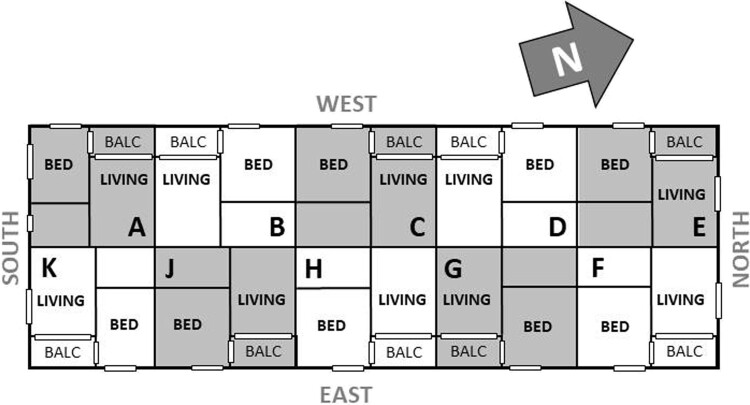
Abstracted building layout

The building consists of four levels, each containing ten one-bedroom apartments with a uniform layout across all floors (see Figure 1; layout abstracted to maintain anonymity). Positioned on an east–west axis, the building’s front aligns with the main road. To mitigate air pollution and noise, east-facing apartments are equipped with mechanical ventilation, while west-facing units rely on natural ventilation. Consistent with typical New Zealand residential practices, the building does not include centralised ventilation or heating/cooling systems. Instead, each apartment features a fixed electric wall panel heater in the living room. In line with the Homestar rating tool requirements, humidity performance (e.g. maintaining a specific ambient RH range) was not explicitly considered in the building's design.

Two IEQ devices (Tether EnviroQs^3^) were installed in each dwelling: one in the bedroom and another in the living room. Devices were mounted on interior walls at standardised heights—1.1 m (seated head height) in living rooms and 1.0 m (sleeping head height) in bedrooms—as recommended by Rosemeier (2014). These consistent locations across all dwellings ensured uniformity, avoided direct sunlight exposure, and minimised disruption to occupants. Practical considerations, such as the study duration and potential obstructions, prevented measurements from the centre of rooms.

The data loggers measured relative humidity (RH) across a range of 0% to 100% with an accuracy of ±2%. Readings were captured every 10 minutes, and the average of the three most recent readings was recorded every 30 minutes, producing two RH readings per hour, 24 hours a day.

Outdoor weather data were sourced from New Zealand’s National Climate Database (CliFlo), which provides long-term climate records and statistical summaries at intervals ranging from ten minutes to daily (Knox et al. 2017). The nearest national weather station is located approximately 8 kilometres from the study site at Auckland’s Museum of Transport and Technology (MOTAT). This station, situated in a similarly urbanised inner-city area, was deemed representative of local climatic conditions. While no significant discrepancies between the weather station and building site data were expected, the influence of Auckland's variable microclimates cannot be entirely excluded, particularly considering the urban heat island effect (Tait et al. 2012).

Data collection spanned three years, focusing on winter months (June, July, and August in the Southern Hemisphere). Results from the thermal comfort and temperature analyses conducted during winter and summer have been published separately. Readings were taken half-hourly from designated rooms in each apartment, yielding approximately 26,496 data points per unit. However, some units recorded fewer readings due to intermittent device issues. A comprehensive analysis revealed:

2020: All data loggers functioned effectively.
2021: Minor reductions in data collection were observed for a few units.
2022: Numerous living room devices failed due to battery issues, limiting the analysis to data from 2020 to 2021.

This extensive dataset enabled a robust evaluation of indoor humidity performance in the monitored dwellings.

AH was calculated for each data record using equations (1) and (2) utilising the pressure, temperature, and RH readings from the data loggers.

(1) $$\mathrm{RH}=\;\frac{P_{i}}{P_{i(\mathrm{sat})}}*100\text{\%}$$

(2) $$\mathrm{AH}=\;\frac{P_{i}*\;MW_{i}}{(P-\;P_{i})*\;MW_{\mathrm{dry}}}$$

Where: Pi is the partial pressure in mmHg

Pi(sat) is the saturation pressure at the temperature in mmHg.

MWi is the molecular weight of water which is 18gH_2_O/ mol.

P is measured by Tether in Pa

MWdry is the average molecular weight of air which is 29gair/ mol.

As the data loggers measure pressure in Pa, a conversion to mmHg was necessary (1 Pa = 0.00750062 mmHg). The AH was then utilised to calculate the dew point using the Magnus formula (Lawrence 2005).

After the winter season in 2022, a meeting was held with residents to solicit consent for participation in semi-structured interviews. Of the forty units, sixteen agreed to participate, with a total of twenty semi-structured interviews conducted in November 2022.

## Results

**Figure 2.**
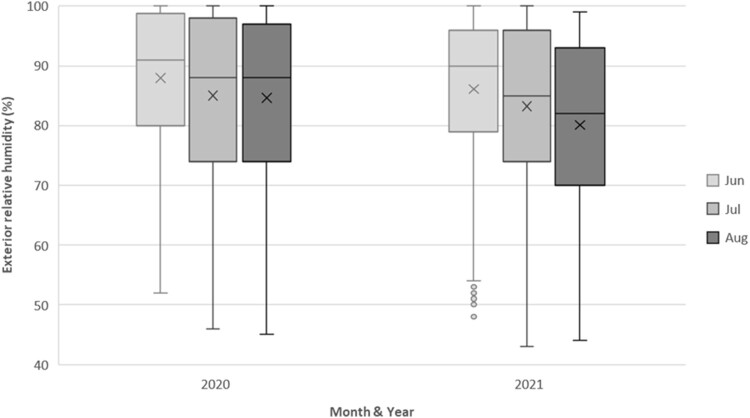
Descriptive statistics for external RH experienced in June, July & August in 2020 and 2021.

Figure 2 depicts the variation in exterior ambient RH levels during the winter seasons of 2020 and 2021, showing consistent exceedance of 60% through both winters.

Table 4 indicates that during the three coldest months of each year, exterior humidity was above RH 60% for over 90% of the time. Given the building's ventilation methods (natural for west-facing units and mixed mechanical/natural for east-facing), the indoor environment was anticipated to reflect the elevated exterior ambient humidity levels.

**Table 4.** Percentage of time spent within different exterior ambient RH ranges in June through August 2020 and 2021.

	% of time spent within ambient RH ranges					
	Exterior ambient RH			Interior ambient RH		
Year	<40%	40% to 60%	>60%	<40%	40% to 60%	>60%
2020	–	3.7	96.3	–	6.5	93.5
2021	–	6.7	93.3	–	10.3	89.7

‘-‘ denotes a value of zero.

Table 4 reveals that the analysed apartments spent only 6.5% and 10.3% of their time within the RH 40% to RH 60% acceptable range over the two studied winters. Conversely, the apartments fell outside the optimal RH range for 93.5% and 89.7% of the time, respectively.

**Figure 3.**
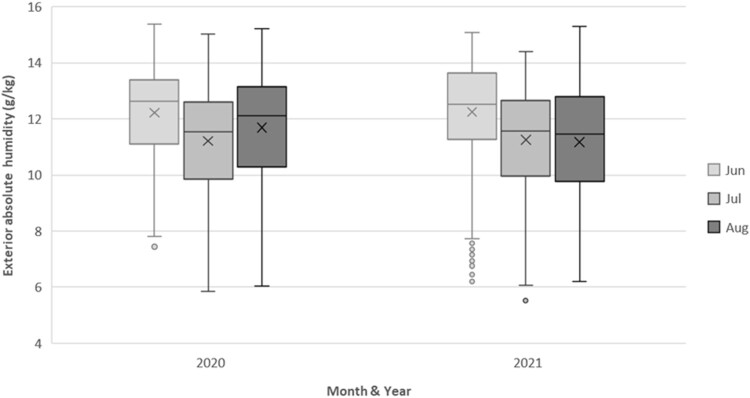
Descriptive statistics for external ambient AH experienced in June, July & August in 2020 and 2021.

Exterior AH exhibited a different pattern (Figure 3). During the 2020/2021 winters, the apartments’ interiors spent 82% to 84% of the time within the 8–14 g/kg range identified in the literature. In contrast, exterior AH fell within this range for only 17% of the time (Table 5).

**Table 5.** Percentage of time spent within different interior ambient AH ranges in June through August 2020 and 2021.

	% of time spent within ambient AH ranges					
	Exterior ambient AH (g/kg)			Interior ambient AH (g/kg)		
Year	< 8	8–14	>14	<8	8–14	>14
2020	5.5	82.6	11.9	15.5	84.3	0.2
2021	7.6	80.0	12.4	17.7	82.1	0.2

Testing the correlation between indoor and outdoor humidities (Table 6) reveals a weak correlation between interior and exterior RH (0.289–0.313) at the 0.01 significance level. In contrast, AH demonstrates a stronger correlation (0.575), aligning with findings in the literature. Introducing a one-hour time lag to account for potential delays in indoor humidity response to outdoor fluctuations does not result in a more robust correlation.

**Table 6.** Pearson correlations for the interior to exterior ambient RH and AH, all units both winters.

	Exterior RH	Ext RH (1hr prior)	Exterior AH	Ext AH (1hr prior)
**Interior RH**	.289**	.313**		
**Interior AH**			.575**	.274**
*N*	930,272	930,272	930,272	930,272

** Denotes significance at the 0.01 level (2-tailed).

* Denotes significance at the 0.05 level (2-tailed).

Analysis of the data by individual unit (Tables 7 and 8) reveals important findings. All units experienced relative humidity (RH) levels exceeding 60% at certain times. The highest-performing units—2D in 2020 and 1J in 2021—spent only 39.2% and 48.1% of their time, respectively, within the recommended RH range of 40% to 60%.

**Table 7.** Percentage of time each dwelling spends within different RH and AH ranges during the 2020 winter.

Unit	Face	% of time spent within ambient humidity ranges					
		Interior ambient RH			Interior ambient AH		
		<40%	40% to 60%	>60%	<8 g/kg	8–14 g/kg	>14 g/kg
0A	W	–	3.5	96.5	3.3	96.7	–
0B	W	–	8.0	92.0	15.7	84.3	–
0C	W	–	3.3	96.7	7.2	92.8	–
0D	W	–	18.9	81.1	8.3	91.6	0.1
0E	W	–	10.8	59.2	5.5	94.5	–
0F	E	–	33.5	66.5	16.2	83.8	–
0G	E	–	3.3	96.7	11.2	88.8	–
0H	E	–	1.8	98.2	18.6	81.4	–
0J	E	–	7.4	92.6	16.5	83.5	–
0K	E	–	1.1	98.9	0.8	99.2	–
1A	W	–	0.1	99.9	0.1	95.2	4.7
1B	W	–	2.4	97.6	–	95.5	4.5
1C	W	–	10.3	89.7	–	90.3	9.7
1D	W	–	3.8	96.2	–	93.0	7.0
1E	W	–	5.3	94.7	–	99.8	0.2
1F	E	–	0.4	99.6	0.2	99.7	0.1
1G	E	–	2.8	97.2	20.2	79.8	–
1H	E	–	–	100	0.5	99.5	–
1J	E	–	30.1	69.9	0.5	99.5	–
1K	E	–	0.5	99.5	2.3	97.7	–
2A	W	–	3.7	96.3	36.7	63.3	–
2B	W	–	6.6	93.4	3.7	96.3	–
2C	W	–	3.5	96.5	3.1	96.9	–
2D	W	–	39.2	60.7	41.0	59.0	–
2E	W	–	18.4	81.5	31.1	68.9	–
2F	E	–	10.5	89.5	39.8	60.2	–
2G	E	–	0.9	99.1	18.1	81.9	–
2H	E	–	7.9	92.1	4.4	95.6	–
2J	E	–	6.3	93.7	11.3	88.7	–
2K	E	–	2.9	97.1	10.2	89.8	–
3A	W	–	1.1	98.9	22.0	78.0	–
3B	W	–	8.3	91.7	43.0	57.0	–
3C	W	–	2.6	97.4	28.2	71.8	–
3D	W	–	3.0	97.0	22.7	77.3	–
3E	W	–	5.2	94.8	6.0	94.0	–
3F	E	–	2.1	97.9	31.4	68.6	–
3G	E	–	2.1	97.9	15.6	84.4	–
3H	E	–	0.4	99.6	10.9	89.1	–
3J	E	–	7.0	93.0	35.5	64.5	–
3K	E	–	2.3	97.7	33.3	66.7	–

**Table 8.** Percentage of time each dwelling spends within different RH and AH ranges during the 2021 winter.

Unit	Face	% of time spent within ambient humidity ranges					
		Interior ambient RH			Interior ambient AH		
		<40%	40% to 60%	>60%	<8 g/kg	8–14 g/kg	>14 g/kg
0A	W	–	4.8	95.2	9.9	90.1	–
0B	W	–	18.6	81.4	25.8	74.2	–
0C	W	–	5.5	94.5	3.9	96.1	–
0D	W	–	5.4	94.6	32.3	67.7	–
0E	W	–	10.4	89.6	7.1	92.9	–
0F	E	–	13.2	86.8	7.3	92.7	–
0G	E	–	18.5	81.5	31.2	68.8	–
0H	E	–	7.5	92.5	27.3	72.7	–
0J	E	–	14.2	85.8	29.5	70.5	–
0K	E	–	0.9	99.1	1.1	98.9	–
1A	W	–	–	100.0	–	94.6	5.4
1B	W	–	–	100.0	–	98.0	2.0
1C	W	–	16.9	83.1	8.9	91.1	–
1D	W	–	3.5	96.5	6.6	93.2	0.2
1E	W	–	2.1	97.9	–	99.6	0.4
1F	E	–	1.5	98.5	0.5	99.3	0.2
1G	E	–	26.7	73.3	20.6	79.4	–
1H	E	–	–	100	2.3	97.6	0.1
1J	E	–	48.1	51.9	10.9	89.1	–
1K	E	–	0.5	99.5	2.9	96.7	0.4
2A	W	–	10.6	89.5	45.8	54.2	–
2B	W	–	18.6	81.4	15.4	84.7	–
2C	W	–	10.9	89.1	5.5	94.5	–
2D	W	–	25.4	74.6	24.0	76.0	–
2E	W	0.1	31.9	67.9	30.3	69.7	–
2F	E	–	18.4	81.6	31.8	68.2	–
2G	E	–	5.3	94.7	21.8	78.2	–
2H	E	–	19.1	80.9	10.6	89.4	–
2J	E	–	15.9	84.1	13.5	86.5	–
2K	E	–	15.6	84.4	14.7	85.3	–
3A	W	–	7.1	93.9	38.1	61.9	–
3B	W	–	5.3	94.7	34.3	65.7	–
3C	W	–	4.0	96.0	27.5	72.5	–
3D	W	–	1.0	99.0	16.0	84.0	–
3E	W	–	3.4	96.6	4.0	95.8	0.2
3F	E	–	1.0	99.0	36.0	64.0	–
3G	E	–	7.5	92.5	18.2	81.8	–
3H	E	–	1.2	98.9	25.1	74.9	–
3J	E	–	1.1	98.9	17.8	82.2	–
3K	E	–	2.9	97.1	37.8	62.2	–

**Figure 4.**
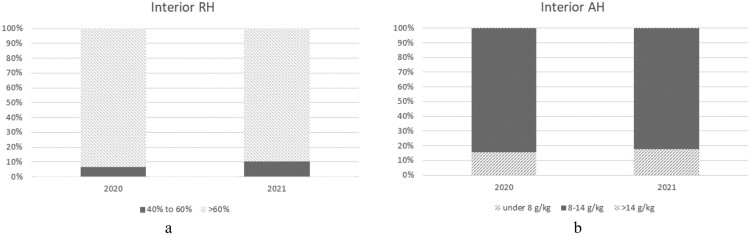
**A**, Cumulative time interior RH spent within RH40% to RH60% range in the winter months of 2020 and 2021. **B**, Cumulative time interior AH spent within the AH 8–14 g/kg range in the winter months of 2020 and 2021.

However, all units consistently maintained absolute humidity (AH) levels predominantly within the 8–14 g/kg range, aligning more closely with recommended indoor air quality standards. This contrast highlights that while the RH analysis suggests potential humidity concerns, the AH assessment indicates a more favourable overall indoor environment (see Figure 4A, B).

Unit 0 K warrants further examination as it was the only unit thermally analysed for the 7-Homestar certification assessment, with its results extrapolated to the rest of the building for certification purposes. Median humidity data for the 2020 and 2021 winters were aggregated by hour for this unit.

**Figure 5.**
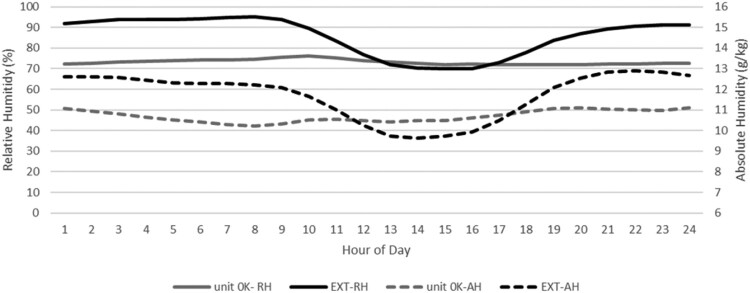
Median RH and AH experienced internally and externally by unit 0 K (the unit assessed for Homestar as the likely worst performing) in June, July & August in 2020.

Figure 5 highlights a deviation in the humidity pattern of Unit 0 K compared to exterior RH and AH. While exterior RH and AH exhibit a pronounced dip between 10:00 am and 5:00 pm daily, Unit 0K's interior humidity does not follow this cycle. Instead, its daily median interior RH and AH values remain stable throughout the day across both winters. Interestingly, exterior RH and AH tend to dip toward Unit 0K's interior values during the day and then rise again each evening and night.

This pattern suggests a potential decoupling of Unit 0K's interior humidity from exterior fluctuations, possibly influenced by factors such as ventilation, occupancy, or thermal mass. Further analysis is necessary to understand these dynamics and their implications for the building's certification assumptions.

The low correlation coefficients (Table 6) may help explain the lack of a clear outdoor 24-h cycle effect observed in Figure 5. Contrary to the expected dip in ambient AH from 10 am to 6 pm, the interior median AH shows a slight increase during these hours, suggesting possible internal moisture generation by occupants. However, the expected peaks in RH and AH during activities such as cooking, washing, and bathing are not prominent. This may indicate that the building’s moisture control measures—including rangehoods, bathroom/laundry extraction systems, and openable windows—effectively manage internally generated moisture.

**Figure 6.**
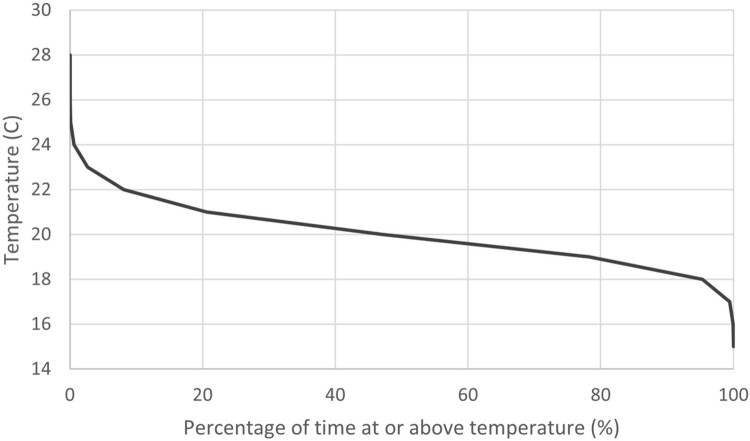
Temperature duration plot for unit 0 K (the unit assessed for Homestar as the likely worst performing) over June, July & August in 2020 & 2021.

**Figure 7.**
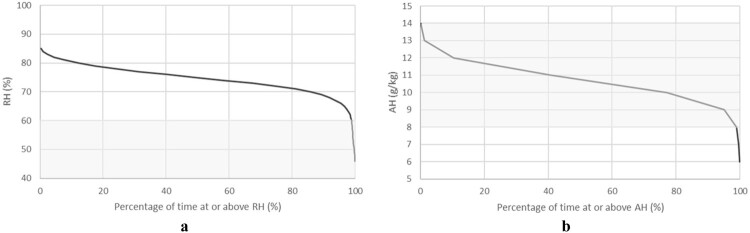
**A**, RH duration plot for unit 0 K (the unit assessed for Homestar as the likely worst performing) over June, July & August in 2020 & 2021. **B**, AH duration plot for unit 0 K (the unit assessed for Homestar as the likely worst performing) over June, July & August in 2020 & 2021.

The duration plots in Figures 6, 7A,B display the percentage of time the interior temperature, RH, and AH remained within specific ranges, providing a cumulative perspective of indoor conditions in Unit 0 K across the winters of 2020 and 2021. These graphs reveal that:

- Temperature consistently remained within the acceptable range of 18°C to 24°C.
- AH was predominantly within the recommended range of 8–14 g/kg.
- However, ambient RH spent the majority of both winters above 60%, exceeding the optimal range for indoor relative humidity.

These findings highlight that, while temperature and AH align with acceptable standards, RH remains elevated, indicating potential concerns for occupant comfort and indoor air quality despite effective moisture control measures.

## Discussion

In this building, high ambient RH dominates throughout the winters, with levels exceeding 60% for 89.7% to 93.5% of the time. This is likely influenced by the predominance of natural ventilation, which allows moist exterior air to enter. However, this explanation is only weakly supported by the Pearson correlation results (r = 0.2–0.3) between interior and exterior RH. These findings are consistent with previous studies by Nguyen et al. (2014), Nguyen and Dockery (2016), which reported minimal correlation between indoor and outdoor RH but identified stronger correlations for indoor and outdoor AH, corroborating the current study’s results.

A building with high RH would generally not be considered ‘dry’. Relative humidity measures the proportion of water vapour in the air relative to the maximum it can hold at a given temperature. High RH indicates air close to saturation, which increases the potential for surface moisture buildup, condensation, and mould growth—typically associated with damp conditions. However, it is possible for AH to be low while RH remains high, especially in colder environments. For example, in winter, even a small amount of water vapour can result in high RH due to the air’s reduced capacity to hold moisture at lower temperatures. In such cases, while the air might feel damp due to elevated RH, the actual moisture content (AH) is low, which can create a perception of dryness, particularly in terms of skin hydration or respiratory comfort.

**Figure 8.**
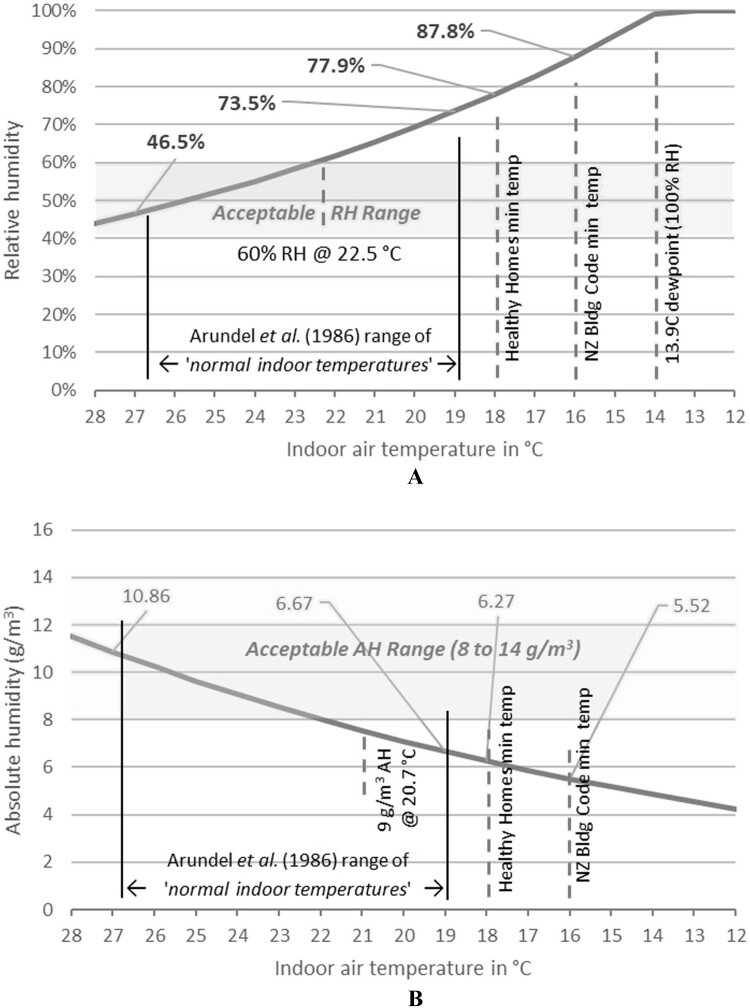
**A**, Relative humidity at 12 g/kg absolute humidity. **B**, Absolute humidity at 50% relative humidity

Figures 8A,B illustrate this relationship graphically. When AH is held constant at 12 g/kg—identified by Qi et al. (2022) as an optimal AH—and temperature varies, a drop to the NZ Building Code minimum temperature of 16°C results in ambient RH rising to 87.8%, exceeding the acceptable range of 40% to 60% as per the Sterling chart. This highlights how low temperatures exacerbate RH levels even when AH remains within optimal limits.

Conversely, if ambient RH is maintained at 50% (the midpoint of the Sterling chart range) and the temperature drops to the NZ Building Code minimum level of 16°C, the corresponding AH becomes 6.8 g/kg. This low AH could increase the risk of viral infections, such as SARS-CoV-2 (Qi et al. 2022). Thus, while a building with high RH would not typically be described as ‘dry’, understanding both RH and AH is critical for accurate assessments of indoor environmental quality.

The finding that this building’s RH levels fall outside the acceptable range, while AH remains within a potentially acceptable range, is particularly relevant for green building rating tools such as Homestar and Green Star. These findings could inform future performance criteria or credits in rating systems.

Reducing RH typically involves increasing temperature, usually through energy-intensive methods such as space heating or dehumidification. Strict adherence to the Sterling chart’s upper RH threshold of 60% could lead to excessive drying of ambient air, increasing residents’ susceptibility to illnesses like influenza or COVID-19, particularly for elderly occupants. Furthermore, this may not reduce risks associated with biotic agents (e.g. mould) or non-biotic pollutants if surface temperatures and RH—key contributors to microbial growth—are not concurrently monitored. Such measures could inadvertently raise energy consumption and health risks, contrary to the objectives of green building initiatives.

Future research should explore the relationship between RH, AH, and health outcomes by correlating humidity metrics with incidences of diagnosed illnesses, such as influenza and COVID-19. Specifically, simultaneous monitoring of both metrics, along with surface-level conditions, could provide a more holistic understanding of the indoor environment.

Although RH levels outside the desired range can signal potential issues with moisture balance, stable AH levels may mask underlying risks. Since RH is temperature-dependent, increasing temperature might lower RH while maintaining sufficient moisture content, potentially obscuring concerns related to comfort, microbial growth, or respiratory health.

To ensure comprehensive evaluations of building performance, both RH and AH should be considered. A potential approach would use RH as a primary performance metric, with AH incorporated selectively—particularly under temperature extremes or health-specific concerns (e.g. viral transmission). This dual focus would align with green building goals by balancing occupant comfort, moisture management, and health outcomes, while minimising energy use and unintended risks.

### Study limitations and recommendations for future research

Based on the collected quantitative data and limited homogeneous qualitative data, this research suggests that while recording and analysing ambient RH is useful, it may not be the sole relevant measure of indoor environmental quality. Ambient AH also appears to offer valuable insights.

Quantitative Data and Occupancy Measurement: This study did not measure real-time occupancy of the dwellings. As noted by Bui et al. (2019), varying occupancy scenarios can significantly influence indoor heat and moisture balance. Future research could be enhanced by incorporating data on actual physical occupancy, enabling a more nuanced understanding of its impact on indoor conditions.

Qualitative Data and Perception Bias: The qualitative component relied on occupant recollections rather than real-time comfort data, which may be influenced by weather conditions at the time of interviews. To address this limitation, future studies could integrate real-time thermal comfort reporting. Expanding the study cohort to include a larger and more heterogeneous sample would also improve the generalizability of findings, moving beyond the current focus on financially disadvantaged individuals aged 65 and older.

Health Outcomes: This study does not incorporate data on resident health outcomes, which could be particularly relevant given the building’s demographic profile. Collaborative research between building science and public health disciplines could investigate the relationship between indoor environmental conditions and health outcomes, providing a more comprehensive understanding of occupant well-being.

External Weather Data: While the use of weather data from a station 8 km away is supported in the literature, placing an external data logger directly on the building could improve the validity of the data by capturing localised conditions more accurately.

By addressing these limitations and expanding the scope of future research, insights into indoor environmental quality, occupant health, and comfort in green-certified buildings can be further refined, contributing to more robust design and operational guidelines.

## Conclusion

This study highlights a critical gap in understanding humidity metrics in green-certified residential buildings and their implications for health, comfort, and energy efficiency. The findings reveal a significant discrepancy between RH and AH as performance indicators. While RH levels in the studied building exceeded recommended thresholds for most of the time, AH levels remained within ranges associated with acceptable indoor environmental quality. This challenges the conventional reliance on RH as the sole metric for evaluating indoor air quality and emphasises the potential value of incorporating AH into building performance assessments, particularly in humid climates.

An important aspect of this research is the ambiguity surrounding the term ‘drier’ in the context of green building performance. Certifications like Homestar claim to deliver ‘drier’ indoor environments but fail to define the term with specificity. This ambiguity allows for varied interpretations, ranging from reduced moisture content (AH) to controlled condensation or ambient RH. Such lack of clarity complicates building performance evaluations and hinders the development of targeted design strategies. As noted by Amaripadath et al. (2023), existing standards and sustainability assessment methods would benefit from including additional humidity indicators. A more holistic definition of ‘drier’ could encompass both RH and AH control, along with strategies to mitigate dampness-related issues such as condensation, moisture intrusion, and microbial growth.

The anecdotal satisfaction of residents with the indoor environment, despite elevated RH levels, underscores the limitations of RH as a standalone measure. This suggests that AH, which quantifies actual moisture content independent of temperature, offers a more nuanced understanding of indoor conditions. The study highlights the importance of aligning ‘drier’ definitions with measurable performance metrics directly related to occupant health, comfort, and energy use. Incorporating both RH and AH into building performance evaluations could address this gap, ensuring buildings achieve both perceived and measurable improvements in indoor environmental quality.

This research suggests that green building rating tools should reconsider their approach to humidity control, moving beyond prescriptive measures to integrate performance-based metrics that account for both RH and AH. Additionally, these tools should explicitly define ‘drier’ using measurable terms to ensure consistent performance outcomes. Future studies should focus on the interplay between RH and AH, examining their impacts on occupant health, energy use, and microbial growth. Expanding research to include diverse building types, climates, and health outcome data could provide a comprehensive understanding of optimal indoor humidity conditions.

By addressing these gaps and clarifying the meaning of ‘drier’, the building industry can enhance the design and certification processes for green buildings, ensuring they deliver healthier, more comfortable, and energy-efficient living environments.

## Notes

1
https://www.usgbc.org/credits/homes-mid-rise/v2008/eqc3

2
https://v2.wellcertified.com/en/performance-rating/thermal%20conditions/feature/2

3
https://www.tether.co.nz/_files/ugd/571de8_1b53d2bf8a4a49eea98562081e066eea.pdf
